# Degradation of lipoxygenase-derived oxylipins by glyoxysomes from sunflower and cucumber cotyledons

**DOI:** 10.1186/1471-2229-13-177

**Published:** 2013-11-09

**Authors:** Danilo Meyer, Cornelia Herrfurth, Florian Brodhun, Ivo Feussner

**Affiliations:** 1 Georg-August-University, Albrecht-von-Haller Institute for Plant Sciences, Department for Plant Biochemistry, Justus-von-Liebig-Weg 11, Göttingen D-37075, Germany

**Keywords:** CoA-ester, Electrophiles, Lipid oxidation, Lipoxygenase pathway, β-oxidation

## Abstract

**Background:**

Oilseed germination is characterized by the degradation of storage lipids. It may proceed either via the direct action of a triacylglycerol lipase, or in certain plant species via a specific lipid body 13-lipoxygenase. For the involvement of a lipoxygenase previous results suggested that the hydroxy- or oxo-group that is being introduced into the fatty acid backbone by this lipoxygenase forms a barrier to continuous β-oxidation.

**Results:**

This study shows however that a complete degradation of oxygenated fatty acids is possible by isolated cucumber and sunflower glyoxysomes. Interestingly, degradation is accompanied by the formation of saturated short chain acyl-CoAs with chain length between 4 and 12 carbon atoms lacking the hydroxy- or oxo-diene system of the oxygenated fatty acid substrate. The presence of these CoA esters suggests the involvement of a specific reduction of the diene system at a chain length of 12 carbon atoms including conversion of the hydroxy-group at C7.

**Conclusions:**

To our knowledge this metabolic pathway has not been described for the degradation of polyunsaturated fatty acids so far. It may represent a new principle to degrade oxygenated fatty acid derivatives formed by lipoxygenases or chemical oxidation initiated by reactive oxygen species.

## Background

During oilseed germination the metabolism of storage lipids serves both, energy and carbon supply for the growing seedling [1]. These storage lipids are deposited in specialized organelles called lipid bodies [2]. In many oilseeds - like sunflower and cucumber - linoleic acid (LA, (9*Z*,12*Z*)-octadeca-9,12-dienoic acid) is a major fatty acid esterified in storage triacylglycerols (TAGs) [3,4]. During germination free fatty acids like LA are released from TAGs by lipid body lipases prior to transport and degradation via β-oxidation in glyoxysomes [5,6]. The *cis*-double bond in LA may constitute a barrier for the conventional β-oxidation cascade and the participation of auxiliary enzymes (Δ^3^,Δ^2^-enoyl-CoA isomerase and NADPH-dependent 2,4-dienoyl-CoA reductase) is necessary for complete breakdown of this fatty acid [7]. Such auxiliary activities have been isolated and characterized first from cucumber and later from Arabidopsis [8-11]. Moreover it was shown, that purified glyoxysomes from sunflower cotyledons can perform the complete degradation of LA to acetyl-CoA in a NADPH-independent way and that this system can be used to follow all sequential reaction steps [12].

Despite this pathway an additional lipoxygenase (LOX)-dependent mobilization of storage lipids has been suggested [13]. The corresponding reaction cascade is initiated by the oxidation of esterified LA by a lipid body specific 13-LOX [14] which is promoted by a lipid body specific patatin-type lipase [15]. This process is accompanied by the formation of lipid oxidation products in the TAG fraction. Up to 20 % of all LA moieties esterified to TAGs where found to be oxygenated in 4-day-old cucumber seedlings [14]. Subsequently, (9*Z*,11*E*,13*S*)-13-hydroperoxy-9,11-octadecadienoic acid (13-HPOD) is released from the TAG fraction, reduced and then it may be transported into the glyoxysomes [13]. The corresponding (9*Z*,11*E*,13*S*)-13-hydroxy-9,11-octadecadienoic acid (13-HOD) serves then as substrate for β-oxidation. Although the 13-LOX mediated mobilization and release of 13-HOD during germination has been well described for sunflower, linseed and cucumber [16], the knowledge on the degradation of this oxylipin remains incomplete. In a previous study it has been suggested, that either the conjugated hydroxy-diene system of 13-HOD or the corresponding electrophilic oxo-diene system of (9*Z*,11*E*)-13-keto-9,11-octadecadienoic acid (13-KOD) cannot be metabolized by the β-oxidation machinery of isolated sunflower glyoxysomes [17]. Although an efficient activation of externally added 13-HOD to 13-HOD-CoA was found using isolated glyoxysomes the data of this study suggested an incomplete turnover of this oxylipin. Only two rounds of β-oxidation occurred with these substrates challenging the hypothesis that lipoxygenation of storage lipids is an additional pathway for storage lipid mobilization.

In the present study the reported protocol for the isolation of glyoxysomes and separation of β-oxidation intermediates from the *in vitro* assays was optimized. Furthermore, the β-oxidation intermediates were characterized by mass spectrometry. Despite intermediates of different chain-length and oxidation states esterified to CoA, different free fatty acids were identified to be released during the degradation process. The data presented show now that a complete degradation of 13-HOD is possible by cucumber and sunflower glyoxysomes, but the system may be sensitive against reactive electrophiles like 13-KOD and its degradation products.

## Results

### LA- and 13-HOD-dependent formation of NADH by glyoxysomal fractions

**Figure 1:**
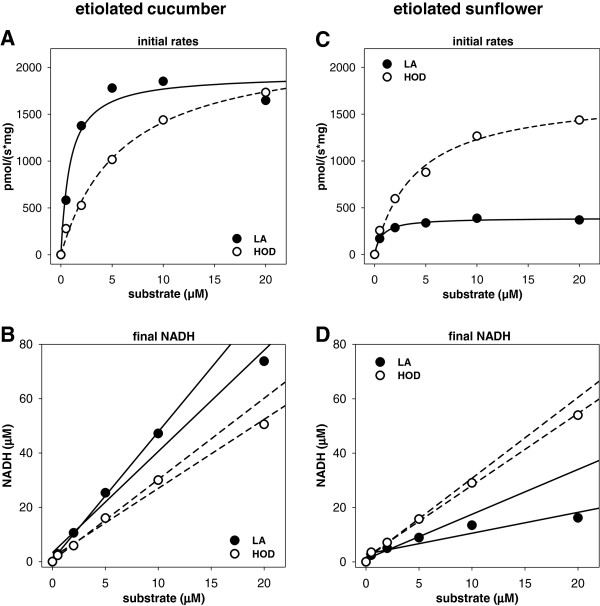
**Turnover of LA and 13-HOD by glyoxysomes from etiolated cucumber (A/B) or sunflower (C/D) in the optical β-oxidation assay.** The figures show one typical experiment while the mean values for all experiments are given in Table 1. **A/C**: Initial rates for NADH-formation from different fatty acid concentrations were fitted according to a hyperbolic equation. Comparable rates of NADH-formation and S_0.5_ values for turnover of 13-HOD by glyoxysomes from cucumber **(A)** or sunflower **(C)** are observed, but only weak rates of NADH-formation from LA by sunflower organelles can be detected. **B/D**: The final concentrations of NADH were calculated from the final absorbance at 340 nm and the formed molecules NADH from each molecule of fatty acid was obtained from the slope of a linear fit. Either all data points or only the concentrations up to 5 μM were used for the fitting procedure. The latter value is less influenced by inactivation effects and is thought to give a more precise result. Again, comparable values for turnover of 13-HOD are obtained for both plants, but NADH formation from LA is strongly decreased when sunflower glyoxysomes are used.

The optical detection of NADH-formation provides a straightforward approach for the determination of both, rate and completeness of fatty acid degradation in case of isolated glyoxysomes where other interfering NADH-forming reactions are absent. Thus, we used this method to determine the preference of glyoxysomal fractions for either LA or 13-HOD. Both substrates give rise to the formation of NADH indicating their activation to the respective acyl-CoA esters and subsequent degradation via β-oxidation. Glyoxysomes from etiolated cucumber or sunflower seedlings show comparable activities towards 13-HOD. While one typical experiment is shown in Figure 1, Table 1 gives the mean values of all experiments. Together, the data suggest, that especially at high substrate concentrations inactivating effects of the applied substrate may have a negative impact on NADH-formation. Therefore, two fits are shown for each plot (Figure 1B and D): The first fit including all data points up to 5 μM results in a steeper slope and thus higher amounts of NADH-formation per substrate. This value most likely describes best the catalytic capacity of the glyoxysomal fractions. For comparison the fit over the whole examined substrate concentration is given as well. As especially the values for substrate concentrations of 10 and 20 μM may be affected by the inactivating effects, these data points do not fit very well to the linear equation. Nevertheless, this plot provides a minimal value for NADH-formation per substrate, which in all cases still supports the finding of more than two rounds of β-oxidation for 13-HOD. Depending on the quality of the glyoxysomal preparation maximal rates of NADH-formation between 1000 and 4000 pmol * s^-1^ * mg^-1^ and S_0.5_ between 4 and 10 μM were found. In both systems the formation of 2.5 - 3.5 molecules NADH from each molecule of 13-HOD was calculated indicating a mean of two to four rounds of β-oxidation for each substrate molecule. This finding shows that an optimized *in vitro* system can proceed up to 4 rounds of β-oxidation in contrast to the previously reported formation of only two rounds [17]. Nevertheless, eight rounds are expected assuming complete degradation.

**Table 1:** Parameters for conversion of LA, 13-HOD and 13-KOD by glyoxysomes from etiolated cucumber or sunflower seedlings in the optical β-oxidation assay

		**Cucumber**	**Sunflower**
**LA**	initial rate (pmol/(s*mg))	1000 – 4500 ^(5)^	400 – 900 ^(3)^
	final NADH (μM/μM LA)	4.7 – 5.6 ^(5)^	1.7 – 2.6 ^(3)^
**13-HOD**	initial rate (pmol/(s*mg))	900 – 3800 ^(5)^	1100 – 2500 ^(5)^
	final NADH (μM/μM HOD)	2.7 – 3.6 ^(5)^	2.2 - 3.2 ^(5)^
**13-KOD**	initial rate (pmol/(s*mg))	624 ^(1)^	780 ^(1)^
	final NADH (μM/μM KOD)	0.95 ^(1)^	1.05 ^(1)^

Parameters were determined as described in Figure 1. Final amounts of NADH are determined for substrate concentrations up to 5 μM. The number of individual experiments with independently purified glyoxysomes are given in brackets.

While no significant differences for the metabolism of 13-HOD by glyoxysomes from cucumber and sunflower were found, both systems show a different behavior towards LA. Using glyoxysomes from cucumber a comparable rate of NADH-formation, but a decreased S_0.5_ value for LA compared to 13-HOD was found. Furthermore, an increased formation of 4.7 – 5.6 molecules NADH from each molecule LA was observed. This points to a higher preference of the cucumber glyoxysomes for the non-oxidized fatty acid LA. However, it should be pointed out that even under these optimized conditions only 6 rounds of β-oxidation can be observed and a quantitative and complete degradation of LA with this *in vitro* system cannot be reached.

Contrary, the organelles from etiolated sunflower possess only weak ability for the formation of NADH from LA. Both, the maximal reaction rate (400–900 pmol * s^-1^ * mg^-1^) and yield of NADH (1.7 - 2.6 μM NADH / 1 μM LA) are decreased significantly when compared to either the conversion of 13-HOD by the same glyoxysomes or to the conversion of LA by glyoxysomes from cucumber.

### Activation of LA

**Figure 2:**
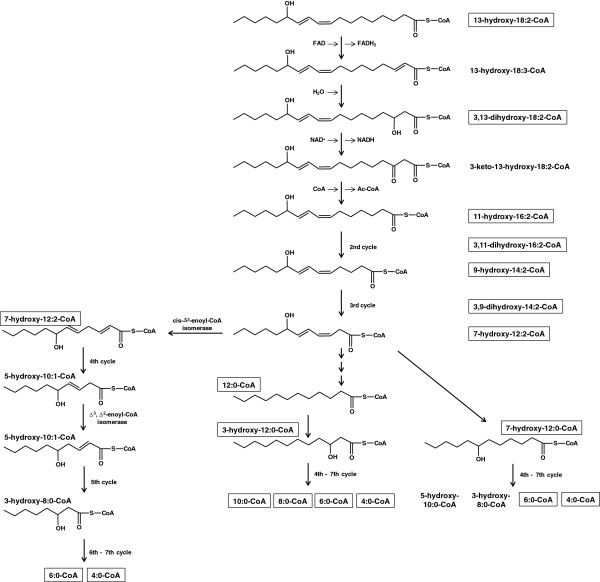
**Possible pathways for degradation of 13-HOD-CoA.** Three rounds of classical β-oxidation reactions yield 7-hydroxy-12:2-CoA as the last intermediate which still contains the initial hydroxy-diene system. The first round of β-oxidation is exemplarily depicted with all expected intermediates. Note that in absence of NAD^+^ the conversion of 13-HOD (or LA) stalls at the 3-hydroxy-intermediate (see Figure 3). Intermediates that have been detected by HPLC or ESI-MS/MS are framed.

**Figure 3:**
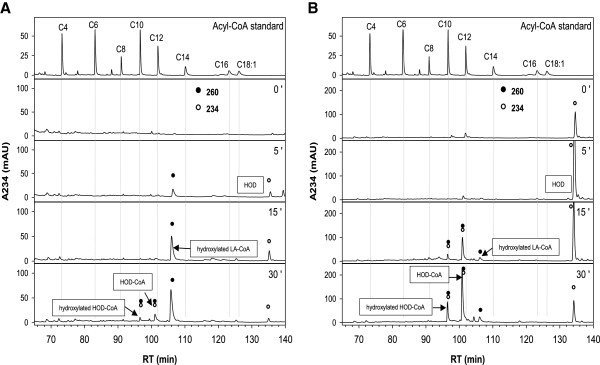
**Time course of activation of LA by glyoxysomes from etiolated cucumber (A) or sunflower (B).** β-oxidation assays were prepared as described in the material and method section with the only exception, that NAD^+^ was excluded from the assay. **A**: Activation of LA by glyoxysomes from cucumber. LA is directly activated to LA-CoA and converted to the 3-hydroxylated form. Small amounts of 13-HOD and 13-HOD-CoA are also observed. **B**: Activation of LA by glyoxysomes from sunflower. LA is rapidly oxidized to 13-HOD which is subsequently activated to 13-HOD-CoA and converted to 3-hydroxy-HOD-CoA. Small amounts of LA-CoA are also detected. HPLC-traces are shown at 234 nm, absorption signals of the individual intermediates are indicated by open (234 nm, conjugated double bond) or closed (260 nm, CoA-ester) circles.

The observation that LA constitutes a good substrate for glyoxysomes from cucumber but not from sunflower raised the question whether this substrate is activated in a comparable way in both systems. In theory, LA can only be activated to LA-CoA and converted to the 3-hydroxy-intermediate when NAD^+^ is excluded from the reaction (as shown for 13-HOD in Figure 2). Indeed, the formation of a distinct compound with an absorption maximum at 260 nm could be observed after 15 minutes when glyoxysomes from cucumber where used (Figure 3A, signals marked by black circles). According to the spectroscopic properties, as well as the retention time, this intermediate was tentatively assigned as 3-hydroxy-LA-CoA. Additional, small amounts of 13-HOD and 13-HOD-CoA were detected (Figure 3A, signals marked by open circles).

However, when glyoxysomes from sunflower where used only minor amounts of the putative 3-hydroxy-LA-CoA were formed after 15 minutes (Figure 3B, signals marked by black circles). Instead, LA was rapidly converted to 13-HOD. Considerable amounts of the oxidized form are already formed in the dead time of this experiment (the time between mixing of the compounds and heat inactivation of the enzymes). 13-HOD rather than LA is then activated to the CoA-ester and further converted to the hydroxylated 13-HOD-CoA derivative (Figure 3B, signals marked by open circles). Thus, LA seems not to be degraded directly by glyoxysomes from sunflower, but a LOX-mediated oxidation may precede its degradation.

### Time resolved profiling of β-oxidation intermediates during 13-HOD degradation

**Figure 4:**
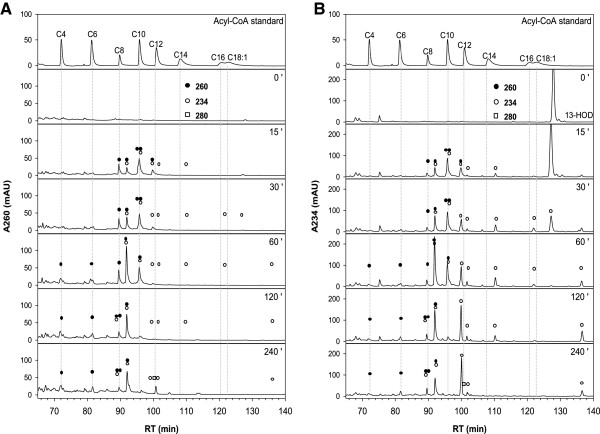
**Time course of turnover of 13-HOD by glyoxysomes from etiolated cucumber.** β-oxidation assays were prepared as described in the material and method section. HPLC-traces after different reaction times were detected at 260 nm **(A)** and 234 nm **(B)**. Absorption signals of the individual intermediates are indicated by open (234 nm, conjugated double bond) or closed (260 nm, CoA-ester) circles and open squares (280 nm, keto-diene system). Besides 13-HOD-CoA (retention time ~ 100 min) acyl-CoAs bearing the conjugated hydroxy-diene system after one (retention time ~ 96 min) and two (retention time ~ 92 min) cycles of β-oxidation are detected and have been validated by MS. The intermediate appearing after 120 min of reaction (retention time ~ 89 min, absorption at 234 and 260 nm) is very likely the 13-HOD-intermediate after three cycles of β-oxidation. Intermediates lacking the 234 nm absorption coelute with authentic acyl-CoA standards with chain length between 4 and 12 carbons. Intermediates lacking the 260 nm absorbtion are presumed to be 13-HOD after one (retention time ~ 111 min) or two (retention time ~ 100 min) rounds of β-oxidation.

**Figure 5:**
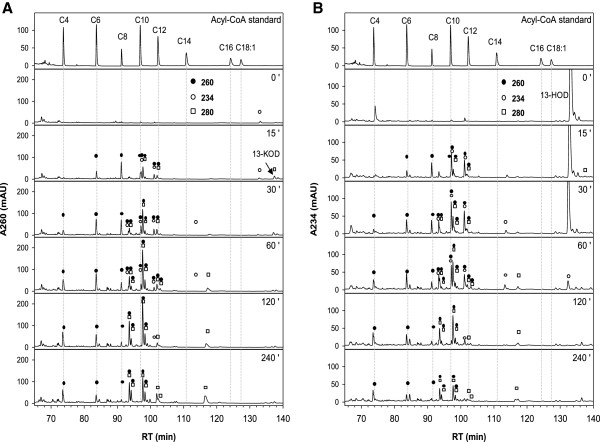
**Time course of turnover of 13-HOD by glyoxysomes from etiolated sunflower.** β-oxidation assays were prepared as described in the material and method section. HPLC-traces after different reaction times were detected at 260 nm **(A)** and 234 nm **(B)**. Absorption signals of the individual intermediates are indicated by open (234 nm, conjugated double bond) or closed (260 nm, CoA-ester) circles and open squares (280 nm, keto-diene system). Besides 13-HOD-CoA (retention time ~ 101 min) acyl-CoAs bearing the conjugated hydroxy-diene system after one (retention time ~ 97 min) and two (retention time ~ 93 min) cycles of β-oxidation are detected and have been validated by MS. The respective oxidized KOD-derivatives elute with slightly elongated retention times (~ 102, 98 and 94 min). Intermediates lacking the 234 nm absorption coelute with authentic acyl-CoA standards with chain length between 4 and 12 carbons.

Previously the ability of glyoxysomes from etiolated sunflower cotyledons to activate 13-HOD to the corresponding acyl-CoA was demonstrated [17]. Even though the β-oxidation intermediates were separated, the identification of many intermediates remained open. Thus, we adopted the reported HPLC-based protocol for the separation of β-oxidation intermediates and adjusted it in order to permit a MS-based identification of distinct species. Furthermore, we attempted to compare the situation in sunflower with cucumber, in which the formation of 13-HOD from storage TAGs has primarily been described [14,15]. 20 μM of 13-HOD were added to a β-oxidation assay containing purified glyoxysomes and all compounds for activation and degradation of fatty acids. After heat inactivation and purification the intermediates were separated by reversed phase (RP)-HPLC in order to allow a time-resolved profiling of 13-HOD degradation (Figures 4 and 5). Additionally, the major compounds were collected from the incubations of cucumber glyoxysomes and identified by ESI-MS/MS (Additional file 1: Figures S3 - S9).

#### Turnover of 13-HOD by cucumber glyoxysomes

13-HOD completely disappears from the reaction mixture containing the cucumber organelles after 60 min (Figure 4B, signal at a retention time of ~ 128 min). Also, the formation of 13-HOD-CoA and the unsaturated intermediates after one or two cycles of β-oxidation (Figure 4B, signals at a retention times of ~ 100, 96 and 92 min) as well as saturated short-chain intermediates were observed (Figure 4A, signals at a retention time of ~ 95, 90, 82 and 72 min). Thus, glyoxysomes from etiolated cucumber bear the general ability for complete degradation of 13-HOD. Additionally, small amounts of an intermediate which harbors the spectroscopic characteristics of the hydroxy-diene system (234 nm) and CoA (260 nm) were detected. Even though the low amounts of this intermediate prevented a MS-based identification, the retention time (~ 89 min) and the spectroscopic properties suggest, that this compound is 7-hydroxy-dodecadienoyl-CoA, the intermediate after three rounds of β-oxidation.

#### Turnover of 13-HOD by sunflower glyoxysomes

Using glyoxysomes from sunflower the general degradation was the same as described by Gerhardt and coworkers [17] (Figure 5):

i. 13-HOD (retention time of ~ 130 min, absorption maximum at 234 nm) is completely consumed after 60 min and activated to 13-HOD-CoA (retention time ~ 100 min, absorption maxima at 234 and 260 nm);

ii. The formation of two degradation intermediates with absorption maxima at 260 and 234 nm was observed (retention times ~ 96 and 92 min);

iii. The appearance of intermediates with absorption maxima at 260 and 280 nm indicating the conversion of the hydroxy-diene- into a keto-diene system was observed.

The intermediates were re-extracted from the HPLC-gradient, and were analyzed by ESI-MS/MS. As suggested before [17], the compounds bearing the 234 nm absorption constitute compounds after one and two cycles of β-oxidation (shown for the metabolites obtained from cucumber, Additional file 1: Figures S6 and S8). Additionally, the identity of the corresponding keto intermediates (13-KOD-CoA and respective intermediates after one and two cycles of β-oxidation) was confirmed. These substances elute with a delay of approximately 1 min regarding their respective hydroxy-derivative. In addition to the previous report short-chain intermediates that lack the typical absorption characteristic for a conjugated diene system were detected (characterized by the absorption of CoA at 260 nm). These intermediates co-elute with authentic standards of saturated acyl-CoAs with chain length between 4 and 12 carbon atoms and could additionally be assigned by ESI-MS/MS as shown for the corresponding incubations with cucumber glyoxysomes (Additional file 1: Figures S4, S7, S9). This finding shows that the hydroxy-diene system in 13-HOD constitutes no general barrier for degradation [18].

Two main differences were observed when cucumber glyoxysomes are compared to the organelles from sunflower (Figure 4 vs. 5): Only traces of intermediates containing the keto-diene system were observed in cucumber. Instead, numerous substances harboring the hydroxy-diene system and having a chain length of 14 or 16 carbon atoms were detected that obviously had lost CoA and thus are very likely released from the β-oxidation cycle (Figure 4B, all signals marked by an open circle and not by a closed circle, between ~ 100 and 122 min).

### Relevance of formation of 13-KOD for the degradation of 13-HOD

The observed formation of distinct amounts of keto-diene intermediates especially by the sunflower organelles (Figure 5, signals marked by an open square) raised the question in how far the oxidation of the hydroxy-diene system is necessary for 13-HOD degradation. Assuming that 13-KOD is an on-pathway intermediate during degradation of 13-HOD one would expect a better or at least comparable formation of NADH when 13-KOD is used as substrate. Actually both, rate and final amount of NADH formation, is decreased when 13-KOD is incubated instead of 13-HOD in a β-oxidation assay containing either glyoxysomes from sunflower or cucumber (Table 1). Thus, 13-KOD is presumably not an essential intermediate for the conversion of 13-HOD. On the contrary, it might be toxic for the β-oxidation machinery since it harbors an electrophilic system that may form Michael adducts with the enzymes involved and thereby inactivating them.

## Discussion

Mobilization of storage lipids is crucial for the development of oil seedlings though it provides energy and carbon supply until autotrophic metabolism is established [1]. Therefore, the degradation of the lipid body phospholipid monolayer by a patatin-type lipase may allow access for different enzymes to the TAG-fraction [15]. The classical pathway of storage lipid mobilization may then be initiated by the TAG lipase-dependent release of free fatty acids like LA from the lipid bodies and subsequent transfer to glyoxysomes where activation to the respective acyl-CoA and β-oxidation takes place [1,6]. Though LA contains two *cis*-double bonds and the canonical β-oxidation reaction cascade only deals with *trans*-double bonds, two additional enzymes (Δ^3^,Δ^2^-enoyl-CoA isomerase and 2,4-dienoyl-CoA reductase) are necessary for its degradation [7].

Besides this classical TAG lipase-initiated pathway, an additional LOX-initiated pathway seems to exist [13]. A lipid body specific 13-LOX oxidizes esterified LA in the TAG-fraction and 13-HPOD is subsequently released from the lipid bodies, reduced to 13-HOD and transported to the glyoxysomes [14]. While the 13-LOX-mediated mobilization and release of 13-HOD during germination has been described for sunflower, linseed and cucumber [16], the knowledge on the degradation of this oxylipin remained incomplete. In a recent *in vitro* study it was shown, that 13-HOD can efficiently be activated to 13-HOD-CoA by glyoxysomes isolated from sunflower seedlings. However, the absence of small chain acyl-CoAs and the apparent stable accumulation of two intermediates suggested a stop of degradation after two rounds of β-oxidation and prompted the authors to the assumption, that the hydroxy-diene system might constitute a general barrier for the β-oxidation machinery [17].

In the present study the protocol for the separation of β-oxidation intermediates was optimized and a methodology for the identification of these compounds has been developed. As reported by Gerhardt and coworkers before [17], the accumulation of two prominent intermediates with apparently higher polarity than 13-HOD-CoA was observed (Figures 4 and 5, retention times ~ 96 and 92 min). Using mass spectrometry the before made assumption that these intermediates are metabolites after one and two cycles of β-oxidation of 13-HOD-CoA was confirmed. Interestingly, these species didn’t constitute the end products of 13-HOD degradation in our experiments. They disappeared with prolonged reaction times and three main classes of other intermediates were detected. Strikingly in both systems, glyoxysomes from cucumber and sunflower seedlings, the formation of short chain acyl-CoAs (saturated acyl-CoAs with chain length between 4 and 12) lacking the hydroxy-diene system were detected. Most importantly, their formation was only detected upon addition of exogenous 13-HOD. This finding clearly demonstrates the general ability of glyoxysomes from these plants for the complete degradation of 13-HOD-CoA.

Moreover, these identified intermediates during 13-HOD degradation allow now to suggest a modified degradation path of this oxylipin (Figure 2, pathway in the middle). Three rounds of classical β-oxidation reactions yield 7-hydroxy-dodecadienoyl-CoA as the last intermediate which still contains the initial hydroxy-diene system. Beside intermediates after the complete first (11-hydroxy-hexadecadienoyl-CoA), second (9-hydroxy-tetradecadienoyl-CoA) and third cycle (7-hydroxy-dodecadienoyl-CoA) also masses that correspond to the respective dihydroxy-intermediates have been detected. They most likely correspond to the 3-hydroxy β-oxidation intermediates of this oxylipin (i.e. third compound from above in Figure 2 and compound with a retention time of ~ 96 min in Figure 3). For the further conversion three pathways are theoretically possible. The first depends on known activities, which successively degrade the hydroxy-diene system in rounds 4 to 6 of β-oxidation (Figure 2, left path) as has been suggested before [13,18,19]. The conjugated hydroxy-diene system of 7-hydroxy-dodecadienoyl-CoA is converted by *cis*-Δ^3^-enoyl-CoA isomerase and Δ^3^,Δ^2^-enoyl-CoA isomerase into classical β-oxidation intermediates. This pathway can be now excluded as it cannot explain the presence of the hydroxy-dodecadienoyl-CoA intermediate and the completely reduced intermediates dodecanoyl-CoA, decanoyl-CoA and octanoyl-CoA that were newly detected in the presented experiments (Figures 4 and 5).

The presence of hydroxy-dodecanoyl-CoA may theoretically be explained, if the conjugated double bond is completely reduced at the C12-stage without conversion of the hydroxy-group at C7 (Figure 2, right path). The resulting 7-hydroxy-dodecadienoyl-CoA could then in principle be further degraded using classical β-oxidation enzymes. However, this alternative route also implies the presence of hydroxylated intermediates with a chain length shorter than C12 and again cannot explain the presence of dodecanoyl-CoA, decanoyl-CoA and octanoyl-CoA. These intermediates can only be explained if the hydroxy-diene system is completely degraded at the C12-stage (Figure 2, middle path). The resulting dodecanoyl-CoA is then completely degraded using normal β-oxidation reactions and is the only path that is supported by our data.

Besides the similar formation of saturated short chain intermediates, the glyoxysomes from cucumber and sunflower differ in the formation of some intermediates. A huge quantity of oxidized free fatty acid intermediates were found during turnover of 13-HOD by glyoxysomes from cucumber indicating an exit from β-oxidation during the first two rounds. Contrary, the transformation of the hydroxy-diene system into a keto-diene system was the major observation when organelles from sunflower were used. Both observations can explain the insufficient formation of only three to four (instead of eight theoretical possible, Figure 1) molecules of NADH from each molecule 13-HOD in the optical assay. The 13-KOD derivatives may be in this way of special interest, as they bear a reactive α,β-unsaturated carbonyl group. Such compounds are termed reactive electrophilic species and this group includes not only fatty acid oxygenation products, but also secondary metabolites, products of hem metabolism, and many others [20]. Reactive electrophilic species like the oxylipins 12-oxo phytodienoic acid and hexenal stimulate the expression of survival genes that are commonly up-regulated during environmental stress and pathogenesis [21]. Formation of keto-dienes has been reported as response to pathogen infection of Arabidopsis leaves, where these compounds may act as phytoalexins [22]. High concentrations of these metabolites may have toxic effects though they inactivate enzymes or change the redox state of the cell due to the reaction with nucleophilic compounds like thiols [20]. Therefore, it is tempting to assume that this inactivation may be the reason for the weak ability of sunflower and cucumber glyoxysomes to degrade 13-KOD directly. This finding also suggests, that 13-KOD is not a necessary on-pathway intermediate for 13-HOD degradation. However, whether formation of reactive electrophilic species from 13-HOD is of relevance *in vivo* and supports seedling viability, or whether it is just artificially enhanced under the applied *in vitro* conditions, remains speculative.

Another striking difference between the cucumber and sunflower system was found for the turnover of LA. Glyoxysomes from cucumber possess a higher preference for the degradation of LA than 13-HOD in the optical assay, whereas the sunflower organelles only show very weak formation of NADH from LA. This finding and the observation, that LA is rapidly converted to 13-HOD and activated to 13-HOD-CoA in absence of NAD, prompt us to assume, that in sunflower LA degradation may be generally initiated by the oxidation to 13-HOD. However, the weak ability of sunflower glyoxysomes to create NADH from LA is somehow enigmatic as the initially formed 13-HOD should be an efficient substrate, but may be restricted to the used sunflower variety. One could even expect an initial burst in NADH-formation, when LA-derived 13-HOD is oxidized to 13-KOD in a NAD-dependent manner. That this conversion indeed depends on NAD can be derived from the observation, that huge amounts of 13-KOD are produced from 13-HOD in a complete β-oxidation assay (Figure 5), but only traces of 13-KOD are formed, when NAD is absent (Figure 3). However, the strong oxidative capacity of the sunflower organelles might explain the low amount of NADH generated during LA turnover, if enzyme inactivation proceeds faster than substrate consumption.

## Conclusions

Together, the presented findings may have implications for the production of unusual fatty acids in plants. Our results may suggest that some plants (like the used sunflower variety) may be faced with severe problems due to their strong oxidative capacity and the resulting conversion of the unusual fatty acids to reactive electrophilic species. Other plants (like cucumber) may be better suited for the production of high amounts of the respective lipids. The herein presented method for the profiling of 13-HOD conversion might serve as a reference system for the classification of currently applied economic plants with strong or weak oxidative capacities. That the here presented activities are not only restricted to seedling germination in the dark is demonstrated by the observation, that 13-HOD can also efficiently be converted by green cucumber seedlings that grew for seven days under light conditions (data not shown). Therefore, it is very likely that the results obtained may be valid for the degradation of oxylipins that are formed during wounding or pathogen infection in the leaf as well [23].

## Methods

### Material

All chemicals, if not mentioned otherwise, were purchased from Sigma–Aldrich (Munich, Germany), Merck (Darmstadt, Germany), or Carl Roth & Co. (Karlsruhe, Germany). All solvents of HPLC grade for the analytical methods were purchased from Acros (Geel, Belgium) or Baker (Griesheim, Germany). Fatty acid substrates were ordered from Cayman Chemicals (Ann Arbor, MI, USA) or Larodan (Malmö, Sweden).

### Plant growth and isolation of glyoxysomes

30 g of seeds from cucumber (*Cucumis sativus*, cv. Chinesische Schlangengurke, N. L. Christensen, Erfurt, Germany) or 40 g of seeds from sunflower (*Helianthus annuus*, cv. Spanners Allzweck, from Hespa Sonnenblumen, Straubing, Germany) were germinated in the dark at 28°C and 100% humidity. Cotyledones were harvested at day 4 (cucumber) or day 7 (sunflower) and glyoxysomes were isolated as described [12]. In brief, cotyledones were homogenized on ice using razor blades in grinding medium (1 M sucrose, 170 mM Tricin pH 7.5, 10 mM KCl, 1 mM MgCl_2_, 1 mM EDTA, 0.7% BSA and 20 mM β-mercaptoethanol) and filtered by miracloth. To remove residual rough plant material the sample was centrifuged at 1500 g for 10 min. The resulting supernatant was subsequently centrifuged at 14000 g for 20 min. The precipitate was resolved in 3 mL of grinding medium and applied to an sucrose density gradient consisting of the following layers: 4 mL 60%, 5 mL 57%, 7 mL 52,5%, 7 mL 47%, 5 mL 43%, 5 mL 35%. Upon centrifugation at 100000 g for 90 min the glyoxysomal fraction was derived from the interphase between 57% and 52,5%. Protein concentration was determined according to Bradford using BSA for calibration and adjusted to 0.15 mg/mL by dilution in 57% sucrose [24].

### Determination of β-oxidation activity

β-Oxidation activity of the glyoxysomal fractions was monitored in an optical assay using the absorption of the arising NADH at 25°C. 100 μL of the glyoxysomal fraction were mixed with 900 μL of a β-oxidation reaction mix and the reaction was initiated by addition of 20 μL of the appropriate fatty acid in ethanol. The final composition of a standard assay was 175 mM Tris pH 8.5, 10 mM ATP, 0.6 mM NAD^+^, 0.6 mM CoA, 20 mM Na-azid, 7.5 mM MgCl_2_, 1 mM DTT and 15 μg/mL glyoxysomal protein.The formation of NADH was quantified using the molar extinction coefficient at 340 nm ϵ_340_ = 6200 M^-1^ * cm^-1^.

### HPLC-based separation of β-oxidation intermediates

1 mL of a β-oxidation reaction mix (see above) was inactivated at distinct time points by incubation at 95°C for 5 min. The samples were purified using solid phase extraction tubes (Strata C18-E, 55 μM, 70A, Phenomenex, Aschaffenburg, Germany) in order to remove sugars and aggregated protein. Therefore, 1 mL sample was applied to a tube equilibrated with 1 mL H_2_O:MeCN:AcOH (95:5:1, by vol.) and subsequently washed with 2 mL of H_2_O:TEA (100:0.25, by vol.). Elution of β-oxidation intermediates was performed with 1 mL Acetonitrile:H_2_O (90:10, by vol.). The organic solvent was evaporated under nitrogen-flow and the remaining sample was adjusted to 120 μL using 50 mM Mes pH 5.0.

100 μL of the sample was subjected to RP-HPLC (column EC 250–2 Nucleosil 120–5 C18; Macherey-Nagel, Düren, Germany) and β-oxidation intermediates were separated using a complex gradient between 25 mM phosphate pH 5.3 and acetonitrile (Additional file 1: Figure S1).

### Identification of β-oxidation intermediates by GC/MS and ESI-MS/MS

The ESI-MS/MS analysis of the intermediates was performed using an Applied Biosystems 3200 hybrid triple quadrupole/linear ion trap mass spectrometer (MDS Sciex, Ontario, Canada) coupled to an ESI chip ion source (TriVersa NanoMate; Advion BioSciences, Ithaca, NY, USA). The respective HPLC fractions were dried under streaming nitrogen, subsequently resuspended in 10 μL of acetonitrile/H_2_O/acetic acid (90:10:0.1, by vol.) and directed to the nanoESI chip adjusted to an ionization voltage of -1.25 kV and a gas pressure of 0.2 psi. The intermediates were ionized in a negative mode and determined in product ion scan mode in a *m/z* range from 350 to 1,100. The collision energy, with nitrogen in the collision cell, was -55 V, declustering potential was -120 V and exit potential was -8 V. The mass analyzers were adjusted to a resolution of 0.7 amu full width at half-height. For each spectrum, 10–20 continuum scans were averaged in multiple channel analyzer mode. The ion source temperature was 40°C, and the curtain gas was set at 10 (given in arbitrary units). For identification of acyl-CoA intermediates, the HPLC fractions were analyzed in precursor ion mode in a *m/z* range from 700 to 1,100 with selected precursor ion of *m/z* 408 [25] and with the same settings as described above.

The GC/MS analysis of the intermediates was carried out using an Agilent 5973 network mass selective detector connected to an Agilent 6890 gas chromatograph equipped with a capillary DB-23 column (30 m × 0.25 mm; 0.25 μm coating thickness; J&W Scientific, Agilent; Waldbronn, Germany). Helium was used as a carrier gas at a flow rate of 1 mL min^-1^. The temperature gradient was 100°C for 1 min, 150–250°C at 8 K min^-1^ and 250°C for 6 min. The injection temperature was set to 220°C. Electron energy of 70 eV, an ion source temperature of 230°C and a temperature of 260°C for the transfer line were used. The ions were recorded in a *m/z* range from 50 to 400.

## Abbreviations

CoA: Coenzyme A; H(P)OD: Hydro(pero)xy octadecadienoic acid; LA: Linoleic acid; KOD: Keto octadecadienoic acid; LOX: Lipoxygenase; MS: Mass spectrometry; NAD: Nicotinamide adenine dinucleotide; RT: Retention time; TEA: Triethylamine; TAG: Triacylglycerol.

## Competing interests

The authors declare that they have no competing interests.

## Authors’ contributions

DM isolated and purified glyoxysomes and performed enzyme assays and incubation experiments. DM, CH, and FB performed the product analysis. DM, FB and IF designed the research and wrote the paper. All authors read and approved the final manuscript.

## Supplementary Material

Additional file 1 Supplemental figures corresponding to “Degradation of lipoxygenase-derived oxylipins by glyoxysomes from sunflower and cucumber cotyledons” by Danilo Meyer, Cornelia Herrfurth, Florin Brodhun and Ivo Feussner. Click here for file

## References

[B1] Graham IA Seed storage oil mobilization Annu Rev Plant Biol 2008 59 1 115 142 10.1146/annurev.arplant.59.032607.092938 18444898

[B2] Hsieh K Huang AHC Endoplasmic reticulum, oleosins, and oils in seeds and tapetum cells Plant Physiol 2004 136 3 3427 3434 10.1104/pp.104.051060 15542496 PMC527141

[B3] Biermann U Friedt W Lang S Lühs W Machmüller G Metzger JO Rüsch gen Klaas M Schäfer HJ Schneider MP New syntheses with oils and fats as renewable raw materials for the chemical industry Angew Chem Int Ed Engl 2000 39 13 2206 2224 10.1002/1521-3773(20000703)39:13<2206::AID-ANIE2206>3.0.CO;2-P 10941055

[B4] Weichert H Kolbe A Kraus A Wasternack C Feussner I Metabolic profiling of oxylipins in germinating cucumber seedlings - lipoxygenase-dependent degradation of triacylglycerols and biosynthesis of volatile aldehydes Planta 2002 215 4 612 619 10.1007/s00425-002-0779-4 12172844

[B5] Eastmond PJ SUGAR-DEPENDENT1 encodes a patatin domain triacylglycerol lipase that initiates storage oil breakdown in germinating Arabidopsis seeds Plant Cell 2006 18 3 665 675 10.1105/tpc.105.040543 16473965 PMC1383641

[B6] Huang AHC Moore TSjr Lipases Lipid Metabolism in Plants 1993 London: CRC Press 473 503

[B7] Goepfert S Poirier Y beta-Oxidation in fatty acid degradation and beyond Curr Opin Plant Biol 2007 10 3 245 251 10.1016/j.pbi.2007.04.007 17434787

[B8] Engeland K Kindl H Purification and characterization of a plant peroxisomal Δ2, Δ3-enoyl-CoA isomerase acting on 3-cis-enoyl-CoA and 3-trans-enoyl-CoA Eur J Biochem 1991 196 699 705 10.1111/j.1432-1033.1991.tb15868.x 2013292

[B9] Engeland K Kindl H Evidence for a peroxisomal fatty acid β-oxidation involving D-3-hydroxyacyl-CoAs. Characterization of two forms of hydro-lyase that convert D-(-)-3-hydroxyacyl-CoA into 2-trans-enoyl-CoA Eur J Biochem 1991 200 1 171 178 10.1111/j.1432-1033.1991.tb21064.x 1879422

[B10] Goepfert S Hiltunen JK Poirier Y Identification and functional characterization of a monofunctional peroxisomal enoyl-CoA hydratase 2 that participates in the degradation of even cis-unsaturated fatty acids in Arabidopsis thaliana J Biol Chem 2006 281 47 35894 35903 10.1074/jbc.M606383200 16982622

[B11] Goepfert S Vidoudez C Rezzonico E Hiltunen JK Poirier Y Molecular identification and characterization of the Arabidopsis Δ3,5, Δ2,4-dienoyl-coenzyme A isomerase, a peroxisomal enzyme participating in the β-oxidation cycle of unsaturated fatty acids Plant Physiol 2005 138 4 1947 1956 10.1104/pp.105.064311 16040662 PMC1183386

[B12] Kleiter AE Gerhardt B Glyoxysomal β-oxidation of long-chain fatty acids: completeness of degradation Planta 1998 206 1 125 130 10.1007/s004250050382

[B13] Feussner I Kühn H Wasternack C The lipoxygenase dependent degradation of storage lipids Trends Plant Sci 2001 6 6 268 273 10.1016/S1360-1385(01)01950-1 11378469

[B14] Feussner I Wasternack C Kindl H Kühn H Lipoxygenase-catalyzed oxygenation of storage lipids is implicated in lipid mobilization during germination Proc Natl Acad Sci U S A 1995 92 25 11849 11853 10.1073/pnas.92.25.11849 11607617 PMC40500

[B15] Rudolph M Schlereth A Körner M Feussner K Berndt E Melzer M Hornung E Feussner I The lipoxygenase-dependent oxygenation of lipid body membranes is promoted by a patatin-type phospholipase in cucumber cotyledons J Exp Bot 2011 62 2 749 760 10.1093/jxb/erq310 21081663 PMC3003817

[B16] Liavonchanka A Feussner I Lipoxygenases: occurrence, functions and catalysis J Plant Physiol 2006 163 3 348 357 10.1016/j.jplph.2005.11.006 16386332

[B17] Gerhardt B Fischer K Balkenhohl TJ Pohnert G Kühn H Wasternack C Feussner I Lipoxygenase-mediated metabolism of storage lipids in germinating sunflower cotyledons and β-oxidation of (9Z,11E,13S)-13-hydroxy-octadeca-9,11-dienoic acid by the cotyledonary glyoxysomes Planta 2005 220 6 919 930 10.1007/s00425-004-1408-1 15526214

[B18] Feussner I Wasternack C Lipoxygenase catalyzed oxygenation of lipids Fett-Lipid 1998 100 4–5 146 152

[B19] Kindl H The oxygen-dependent modification of triacylglycerols and phospholipids, the different way of initiating lipid body mobilization Z Naturforsch 1997 52c 1–2 1 8

[B20] Farmer EE Mueller MJ ROS-mediated lipid peroxidation and RES-activated signaling Annu Rev Plant Biol 2013 64 1 429 450 10.1146/annurev-arplant-050312-120132 23451784

[B21] Davoine C Falletti O Douki T Iacazio G Ennar N Montillet J-L Triantaphylides C Adducts of oxylipin electrophiles to glutathione reflect a 13 specificity of the downstream lipoxygenase pathway in the tobacco hypersensitive response Plant Physiol 2006 140 4 1484 1493 10.1104/pp.105.074690 16500992 PMC1435824

[B22] Vollenweider S Weber H Stolz S Chetelat A Farmer EE Fatty acid ketodienes and fatty acid ketotrienes: michael addition acceptors that accumulate in wounded and diseased Arabidopsis leaves Plant J 2000 24 4 467 476 10.1046/j.1365-313x.2000.00897.x 11115128

[B23] Mosblech A Feussner I Heilmann I Oxylipins: Structurally diverse metabolites from fatty acid oxidation Plant Physiol Biochem 2009 47 6 511 517 10.1016/j.plaphy.2008.12.011 19167233

[B24] Bradford MM A rapid and sensitive method for the quantitation of microgram quantities of proteins utilizing the principle of protein-dye binding Anal Biochem 1976 72 248 254 10.1016/0003-2697(76)90527-3 942051

[B25] Hankin JA Murphy RC MALDI-TOF and electrospray tandem mass spectrometric analysis of fatty acyl-CoA esters Int J Mass Spec 1997 165 467 474

