# Supplementary material for: Degradation of lipoxygenase-derived oxylipins by glyoxysomes from sunflower and cucumber cotyledons
Source: BMC Plant Biol. 2013 Nov 9;13:177. doi: 10.1186/1471-2229-13-177 (PMC3831820; doi:10.1186/1471-2229-13-177)
Supplement: Additional file 1 — Supplemental figures corresponding to “Degradation of lipoxygenase-derived oxylipins by glyoxysomes from sunflower and cucumber cotyledons” by Danilo Meyer, Cornelia Herrfurth, Florin Brodhun and Ivo Feussner. [file 1471-2229-13-177-S1.docx]

Supplemental figures corresponding to “Degradation of lipoxygenase-derived oxylipins by glyoxysomes from sunflower and cucumber cotyledons” by Danilo Meyer, Cornelia Herrfurth, Florin Brodhun and Ivo Feussner

Supplemental Figure S1: HPLC gradient for the separation of β-oxidation intermediates. The complex gradient between A (25 mM phosphate, pH 5.3) and B (acetonitrile) is shown in the lower panel and the table (right panel). β-Oxidation intermediates are separated between 75 and 130 min as indicated in the upper panel with authentic acyl-CoA standards. CoA and acetyl-CoA elute with retention times between 55 and 65 min.

Supplemental Figure S2: Fragmentation pattern of acyl-CoAs during MS/MS as given in the product spectra in Supplemental Figures S4 – S9.

Supplemental Figure S3. Preparative HPLC-profile for turnover of 13-HOD by glyoxysomes from etiolated cucumber. A β-oxidation assay (1 h incubation) was prepared as described in materials and methods with the only exception that a 6 mL reaction was used instead of 1 mL. Absorption traces for 260 nm (solid line, indicating CoA) and 234 nm (dashed line, indicating conjugated hydroxy diene system) are shown. Peaks 1 to 6 were collected, evaporated under nitrogen-flow and solved in 10 µL acetonitrile:water:acetic acid (90:10:0.1) before ESI-MS analysis (see section 2.4). The identified intermediates (Supplemental Figures S4 – S9) are arranged in the upper panel according to their chemical composition.

Supplemental Figure S4: ESI-MS spectra for peak 1 (RT 89.1 – 89.9 min). Precursor ion analysis focusing on CoA-esters (m/z 408, left) and the respective MS/MS spectra from product ion analysis of octanoyl-CoA (m/z 892, right) from a β-oxidation assay with glyoxysomes from etiolated cucumber and 13-HOD after 1 h incubation.

Supplemental Figure S5: ESI-MS spectra for peak 2 (RT 90.7 – 91.3 min). Precursor ion analysis focusing on CoA-esters (m/z 408, left) and the respective MS/MS spectra from product ion analysis of dihydroxy hexadecadienoyl-CoA (m/z 1032, right) from a β-oxidation assay with glyoxysomes from etiolated cucumber and 13-HOD after 1 h incubation. Dihydroxy hexadecadienoyl-CoA is one of the intermediates during the second round of β-oxidation.

Supplemental Figure S6: ESI-MS spectra for peak 3 (RT 91.3 – 92.3 min). Precursor ion analysis focusing on CoA-esters (m/z 408, left) and the respective MS/MS spectra from product ion analysis of hydroxy tetradecadienoyl-CoA (m/z 988, right) from a β-oxidation assay with glyoxysomes from etiolated cucumber and 13-HOD after 1 h incubation. Hydroxy tetradecadienoyl-CoA is the intermediate after two rounds of β-oxidation.

Supplemental Figure S7: ESI-MS spectra for peak 4 (RT 94.0 – 94.6 min). Precursor ion analysis focusing on CoA-esters (m/z 408, upper left) and the respective MS/MS spectra from product ion analysis of decanoyl-CoA (m/z 920, lower left), hydroxy dodecanoyl-CoA (m/z 964, upper right) and dihydroxy octadecadienoyl-CoA (m/z 1060, lower right) from a β-oxidation assay with glyoxysomes from etiolated cucumber and 13-HOD after 1 h incubation. Either 3-hydroxy dodecanoyl-CoA or 7-hydroxy dodecanoyl-CoA can account for the compound with m/z 964. Dihydroxy octadecadienoyl-CoA is one of the intermediates during the first round of β-oxidation.

Supplemental Figure S8: ESI-MS spectra for peak 5 (RT 94.6 – 96.4 min). Precursor ion analysis focusing on CoA-esters (m/z 408, left) and the respective MS/MS spectra from product ion analysis of hydroxy hexadecadienoyl-CoA (m/z 1016, right) from a β-oxidation assay with glyoxysomes from etiolated cucumber and 13-HOD after 1 h incubation. Hydroxy hexadecadienoyl-CoA is the intermediate after one round of β-oxidation, and its accumulation was supposed by Gerhardt and coworkers. Note that the compound with m/z 1014 also coelutes in peak 5. This mass indicates the presence of the corresponding keto derivative of hydroxy hexadecadienoyl-CoA and was found to a much higher content in assays containing glyoxysomes from sunflower.

Supplemental Figure S9: ESI-MS spectra for peak 6 (RT 100 – 100.7 min). Precursor ion analysis focusing on CoA-esters (m/z 408, left) and the respective MS/MS spectrum from product ion analysis of dodecanoyl-CoA (m/z 948, right) from a β-oxidation assay with glyoxysomes from etiolated cucumber and 13-HOD after 1 h incubation. Note that dodecanoyl-CoA constitutes the intermediate with the longest chain length that lacks the complete hydroxy diene system. The compound with m/z 1044 corresponds to traces of 13-HOD-CoA.
